# Home (Office) is where your Heart is: Exploring the Identity of the ‘Corporate Nomad’ Knowledge Worker Archetype

**DOI:** 10.1007/s12599-023-00807-w

**Published:** 2023-04-11

**Authors:** Julian Marx, Stefan Stieglitz, Felix Brünker, Milad Mirbabaie

**Affiliations:** 1 School of Computing and Information Systems, The University of Melbourne, Melbourne, Australia; 2 Chair of Business Information Systems and Digital Transformation, University of Potsdam, Potsdam, Germany; 3 Department of Information Systems, Paderborn University, Paderborn, Germany

**Keywords:** Corporate nomadism, Identity theory, Home office, Knowledge work, Digital nomadism

## Abstract

Working conditions of knowledge workers have been subject to rapid change recently. Digital nomadism is no longer a phenomenon that relates only to entrepreneurs, freelancers, and gig workers. Corporate employees, too, have begun to uncouple their work from stationary (home) offices and 9-to-5 schedules. However, pursuing a permanent job in a corporate environment is still subject to fundamentally different values than postulated by the original notion of digital nomadism. Therefore, this paper explores the work identity of what is referred to as ‘corporate nomads’. By drawing on identity theory and the results of semi-structured interviews, the paper proposes a conceptualization of the corporate nomad archetype and presents nine salient identity issues of corporate nomads (e.g., holding multiple contradictory identities, the flexibility paradox, or collaboration constraints). By introducing the ‘corporate nomad’ archetype to the Information Systems literature, this article helps to rethink established conceptions of “home office” and socio-spatial configurations of knowledge work.

## Introduction

In recent years, the knowledge work sector has undergone a major transformation (Ghislieri et al. 2018; Richter et al. 2018; Wang et al. 2020). In the early 2000s, the typical knowledge worker was connoted with a corporate 9-to-5 employee who was paid for thinking while being in an office building (Davenport 2005). Since then, the widespread implementation of information and communication technologies (ICT) has enabled decentralization and remote work (Hafermalz and Riemer 2016). Thus, knowledge workers are now increasingly able to work from *private places* such as a home office. In parallel, the niche phenomenon of digital nomadism has emerged among knowledge workers who prioritize location independence, work autonomy, and lifestyle over career opportunities (Richter and Richter 2020). More recent work in this research stream, however, has observed a mainstreamization of digital nomadism due to various factors, such as advancing digitalization and the COVID-19 pandemic, which forced corporations to soften the rigid rules of 9-to-5 working models (Aroles et al. 2020; Frick and Marx 2021; Wang et al. 2020). This development demonstrates that the notion of *working from a private place,* such as a home office, is not necessarily bound to a physical location. Thus, the idea of *home office* must allow an interpretation that separates it from a permanent residence and other physical objects (e.g., workstations, devices, and work equipment). Consequently, we acknowledge that knowledge workers can leverage ICT to a degree where they create their *private place* wherever they feel comfortable and productive (Ajzen and Taskin 2021). Therefore, this paper challenges the historically grown understanding of the *home office* concept, which binds it to a physical location and objects (Lal et al. 2021).

The rising number of hundreds of thousands of knowledge workers engaging in new work practices has led to an increased overlap between digital nomadism and corporate structures (Frick and Marx 2021; Schlagwein 2019). Furthermore, recent research suggests that knowledge work will continue to incorporate more traits of the digital nomad archetype and that the changes we are facing post-COVID-19 may be *"the dawn of a new era of knowledge work”* (Wang et al. 2020, p. 1). Whereas early research found that only a small percentage of digital nomads was employed in large companies (Andrade et al. 2013; Müller 2016), corporate employees now push more toward location independence. While around five percent of corporate employees worked location independently before 2020, during the pandemic, that number has risen to 30 percent (Levanon 2020). This development yields a notable research problem because, on a conceptual level, what we know about the digital nomad archetype is based on work identities revolving around freelancing, entrepreneurship, or gig work. Digital nomadism and corporate work were practically incompatible because of conflicting values between corporate management and hyper-individual work identities (Kong et al. 2019).

Where corporate knowledge work used to be defined by a Taylorist paradigm involving the physical presence and strict work schedules, it has become much more independent from time and place (Ghislieri et al. 2018; Wang et al. 2020). One manifestation of this development can be found in the phenomenon we refer to as *corporate nomadism*, that is, corporate employees practicing digital nomadism. One example for the emergence of corporate nomadism is the rapid influx of thousands of knowledge workers flooding the Canary islands for long-term stays (Vega 2020).

We argue that this phenomenon is worthwhile to be examined closer as it raises issues on various individual and organizational levels. In this paper, we focus on corporate nomads' individual work identity formation and how it differs from that of digital nomads and 9-to-5 corporate workers. As corporate nomadism occurs at the intersection of the individual, the organization, and technology, it is of vital interest to the IS community (Richter and Richter 2020).

We draw on identity theory to better understand how corporate nomads develop a new self-concept regarding their work (Burke and Stets 2009). In this context, we assume that roles and relationships influence the identity of corporate nomads. Those relationships and role expectations, e.g., among colleagues, may affect an individual's thinking and behavior toward others. Moreover, individuals develop and maintain many identities within different personal, social, and material contexts. These are validated through an identity verification process of investment, self-esteem, and rewards (Burke and Stets 2009; Stryker and Burke 2000). A corporate nomad, for example, must make certain investments, such as long-term travel or increased self-organization, to be able to obtain an aspired identity.

The combination of corporate values and those of digital nomadism, however, can lead to discrepancies within the self-concept of a corporate nomad. One example is the dependency on ICT to connect with colleagues when it comes to coordinating work. Another challenge arises between the conflicting priorities of individual freedom to choose workplace and hours and still being part of fixed corporate structures (Frick and Marx 2021). This tension can affect the identity of individuals, including their thinking and behavior (Burke and Stets 2009; Orlikowski and Scott 2021). Since corporate nomadism is an emerging phenomenon, it is crucial to understand how corporate nomads form their identity and what issues they deal with when doing so. This leads to the following research questions:

**RQ 1**. How* can a corporate nomad archetype be conceptualized?*

**RQ 2.** *What are potential identity issues of corporate nomads?*

To answer these research questions, we conducted fourteen semi-structured qualitative interviews with corporate nomads. The results were analyzed with a structuring content analysis, as suggested by Kuckartz (2014). The data was deductively categorized through the lens of identity theory to better understand the corporate nomad archetype (RQ1) and approached abductively to unearth potential identity issues (RQ2). This paper contributes to IS literature in a threefold manner. First, this paper defines the corporate nomad archetype and demarcates it from existing knowledge worker archetypes such as the corporate 9-to-5 worker and the digital nomad. This is important because digital nomadism rests upon characteristics such as autonomy and independence that unfold outside of corporate boundaries. Considering this tradition of the digital nomad concept in IS literature, it does not serve as a useful analytical unit for researchers interested in nomadic behavior in corporate contexts. Against this backdrop, our work advances the scientific debate by delineating the conceptual nuances of corporate workers, digital nomads, and corporate nomads. Second, we contribute to identity theory by showing how mobile ICT use (exemplified by corporate nomad work practices) impacts the formation of individual work identities. In this context, this study reveals how investments, self-esteem, and rewards relate to work identity dimensions such as space, time, and control. Third, by identifying potential identity issues of corporate nomads, we derive new knowledge that informs corporate management and nomads about how to overcome discrepant conceptions of working in a *home office* and how to implement corporate nomad work for competitive and individual advantage. With these contributions, this work aims to mark a first step to refining the conceptual breadth of the debate about the future of knowledge work at the intersection of individual work autonomy, institutionalism, and technology.

In the following chapters, the current spectrum of knowledge worker archetypes is described, followed by an explanation of identity theory as well as potential issues that have been part of the debate in IS research. Lastly, we explain our methodological approach, report our findings, and discuss them. Conclusively, a summary, limitations, and recommendations for further research are provided.

## Theoretical Background

### Individual Knowledge Worker Archetypes

To work towards a conceptualization of the corporate nomad, we draw on definitions of established knowledge worker archetypes. The corporate nomad can be understood as a neologism consisting of two already existing knowledge worker archetypes: the 9-to-5 corporate worker and the digital nomad.

The traditional 9-to-5 corporate worker is typically a full-time employee who is present at a corporation throughout the bulk of her working hours. Corporate 9-to-5 workers complete their tasks responsibly while tracking time and accepting the possibility of overtime (Wilk 2016). Therefore, they are very limited in terms of their own organization at work, which is characterized by control and detailed records of work performed on behalf of the corporation (Wang et al. 2020). Accordingly, the place of work is also defined quite rigidly: typically, by an office building in city centers. This impacts work-life balance to a considerable extent, especially regarding overtime, high rents in cities, or commuting times from the suburbs, resulting in corporate workers sacrificing free time for work (Wang et al. 2020; Wilk 2016). Corporate workers are not unaffected by digital transformation and use technologies for more optimal cooperation, organization, and performance (Ghislieri et al. 2018), though the focus while working with technology is on precision and standardized procedures and less on freedom and creativity (Wang et al. 2020). As a result of their full-time – and often permanent – employment, corporate workers generally possess a relatively strong safety net. They have access to benefits such as payment during illness or parental leave (Prester et al. 2019).

Across the spectrum of knowledge worker archetypes, the digital nomad exemplifies an alternative conception of work in comparison to the 9-to-5 corporate worker archetype. The work of digital nomads does not tie them to a corporate space – since they are usually freelancers or entrepreneurs (Schlagwein and Jarrahi 2020). Moreover, they make use of mobile ICT to be able to travel to different places while working at the same time (Kong et al. 2019; Lee et al. 2019). Digital nomads decide for themselves when and where they work, often merging leisure time with worktime, which blurs work-life boundaries (Wang et al. 2020). They value the freedom to use ICT of their choice that enable a lifestyle combining working, travelling, and shaping their social network in parallel (Kong et al. 2019; Lee et al. 2019; Prester et al. 2019). Digital nomads’ work is based on their ability to give and receive in an engaging community with other digital nomads (Jiwasiddi et al. 2022; Prester et al. 2019). Their social safety net is based on independent financial provision, the country they reside in, but also the size of their client base and social network (Wang et al. 2020), which makes networking an essential part of their lives (Vidaillet and Bousalham 2018).

Interestingly, way before the popularization of the digital nomad movement, Chen and Corritore (2008) reported a supposed positive relationship between organizational support for nomadic behavior and employee job satisfaction. This was an early indicator of the potential impact of the nomadic behavior cultivated in corporate environments we witness today. Nonetheless, more recent literature still reports general skepticism about such cultural changes in most branches. Within the last decade, an exhaustive acceptance of digital nomad working models in the corporate sector never really went beyond several IT companies granting software developers remote work arrangements (Schlagwein 2018; Marx et al. 2021). However, not acknowledging the apparent change in individual preferences of knowledge workers, exemplified by recent observations of corporate nomadism, may cause earnest issues for corporations. First, the increasing demand for knowledge workers intensifies the ‘war for talent’ across sectors and not only the IT industry. Consequently, organizations are pressured to adapt their working models toward the demands of highly skilled knowledge workers. Second, a large increment in flexible and digital working models may challenge the organization of work altogether, as physical meetings, office space, or company cars become superfluous. The primary reason for the problems of digital nomad corporate work, according to Kong et al. (2019), is a matter of conflicting values. A lack of understanding of each other’s preferences, poor implementation, or adherence to their respective institutional logics and misalignment between the two worldviews so far have impeded the successful association of both ideals^1^ of work.

However, IS research has recently begun to provide evidence that corporate nomadism is indeed a viable form of knowledge work. Frick and Marx (2021) found that corporate managers acknowledge the potential value of digital nomad work within their teams and regard employee satisfaction, enforced digitization, and potential economic gains as motivators for corporate nomadism. In another study, Ajzen and Taskin (2021) present the compelling argument that increasingly flexible working models, such as nomadic behavior foster the emergence of a new and collective (spatial) identity in corporate workspace. The authors observed that office spaces are changing, with a lot of organizations massively scaling down physical office space. Therefore, practicing corporate nomadism (as one example of what the authors call “flexwork”) is a necessary means in order for organizations to achieve a collective understanding of what “space” is and how much of it is offered by an organization. Table 1 summarizes the hitherto presented concepts of individual knowledge work that inform the corporate nomad archetype.

**Table 1:** Juxtaposition of 9-to-5 corporate worker and digital nomad archetype

Dimension	Knowledge worker archetype			Supporting literature
	9-to-5 Corporate worker	Digital Nomad		
Control	High organizational control	Self-organization and self-leadership	e.g., Wilk (2016) and Wang et al. (2020)	
Time	Fixed time schedules	Flexible; work-life blending	e.g., Schlagwein (2018) and Frick and Marx (2021)	
Social Environment	Stability; social safety net	Dependency on environment; constant networking	e.g., Lee et al. (2019) and Vidaillet and Bousalham (2018)	
Technology	Standardized ICT use	Unconstrained technological choices	e.g., Ghislieri et al. (2018) and Kong et al. 2019	
Space	Fixed, immobile office space, company-owned	Fluctuating; shared (e.g. co-working spaces) or rented spaces (e.g. hotel rooms)	e.g., Ajzen and Taskin (2021)	

### Identity Theory

Corporate nomads aiming to work locally independent but socially interdependent represent a new intertwinement of corporate work and digital nomadism. As part of that phenomenon, it is essential to conceptualize an identity construct to define corporate nomads and identify potential identity issues they encounter. We prefer the concept of ‘identity’ of corporate nomads at an individual level to stress the impact of networks of roles and relationships that they are embedded in, which in turn influence the role, person, thinking, and behavior of individuals relative to others (Burke and Stets 2009; Stryker and Burke 2000). At an individual level, forming an identity is a process impacted by multiple factors, therefore, developing role, personal and material identities is inevitable to form a self-concept. Role identity (e.g., parent, co-worker, etc.) refers to internalized expectations associated with performing a role (Burke and Stets 2009). With corporate nomads, a shift in work-related roles can be expected, thus a change in their work identity, since they are not physically embedded in an organizational environment with traditional and hierarchical structures anymore (Prester et al. 2019). Furthermore, Alvesson and Willmott (2002) state that *“when an organization becomes a significant source of identification for individuals, corporate identity (the perceived core characteristics of the organization) then informs (self-)identity work”* (p.625). However, emotions may impact the formation of corporate nomads’ professional role identity and vice versa by applying distinct emotion management strategies that are directly associated with the perceived role identity or the organizational culture (Winkler 2018). Personal identity (e.g., an athletic or artistic person identity) refers to the characteristics, values, and norms that individuals chose to make them unique compared to other entities (Stets and Serpe 2013). Another identity that became an even greater focus of attention is material identity and identity as a new form. Material identity refers to material objects individuals are bound to and see as integral to the self-concept (Carter and Grover 2015). However, material objects can also be understood as distinct types of software such as ICT or artificial intelligence (Mirbabaie et al. 2022, 2021). With rapid technological advancement and a trend towards pervasive computing, working conditions have changed drastically and created a new form of economic activity, especially for corporate nomads. Between using ICT to profit from workplace flexibility, on the one hand, and relying on ICT on the other hand, interactions with ICT are essential for corporate nomads’ work and form an integral part of their self-concept (Reichenberger 2017).

Extant work on the role of ICT for (mobile) corporate knowledge workers may provide additional insights to underpin the identity formation processes of corporate nomads. In this context, research from the field of computer-supported cooperative work (CSCW) has examined how changing space and time dimensions impact work processes. In the context of digital nomads, too, the workplace may be *“both a practical concern as well as an outcome of their strategies to enable their work to proceed”* (Humphry 2014, p.187). D’Andrea and Gray (2015) emphasize that work flexibility that emerged through using mobile ICT was closely tied to economic motivators. As ICT in the knowledge economy create a fertile soil for growing knowledge-intensive organizations, dynamic and diverse mobile practices emerge. However, the authors also highlight that those developments lead to *“intensified work rhythms and controls, undermining work-life balance”* (p.103). In terms of the organizational control of knowledge workers, Alvesson and Willmott (2002) argue the identity of employees is essential due to the differentiation of cultural, economic, and self-image elements. The process of constructing an individual identity at work also referred to as the concept of *identity work*, might be fluid, unstable, and reflexive (Brown 2015).

**Fig. 1:**
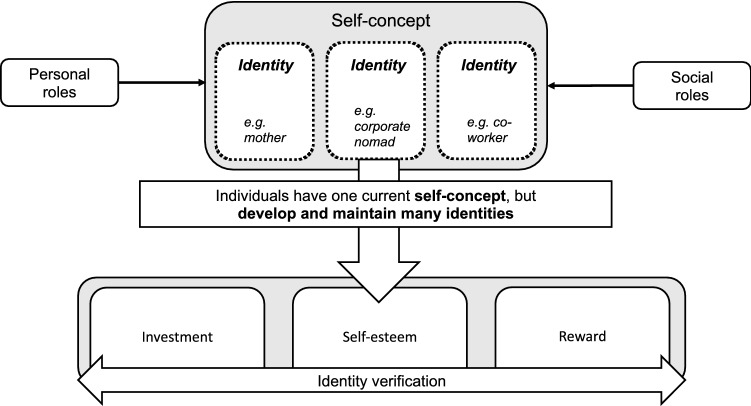
Illustration of the identity formation process based on McCall and Simmons (1978), Stets and Burke (2005), and Burke and Stets (2009)

However, there is not a simple answer to the question “who am I?” as individuals hold many identities. Therefore, deciding on an identity and defining which one is more important in certain contexts is a key pursuit of individuals. Identity theory, against this backdrop, aims to provide a conceptual framework to understand this process (Stets and Serpe 2013). Generally, individuals tend to keep the perception of others congruent with how they see themselves (Burke and Stets 2009). Consequently, whenever an identity becomes the focus of behavior, individuals invest in keeping their perceptions of how others view them congruent with meanings held by them and their identity (Carter and Grover 2015). But once they perceive discrepancies, individuals try to change the environment or their behavior. This is referred to as an *investment* in identity. In the second step, when congruency is achieved, the prevailing identity is verified, and *self-esteem* is protected or enhanced. Enhanced self-esteem, in turn, triggers intrinsic and extrinsic *rewards*, reinforces the identity, and encourages further identity-verification attempts (Burke and Stets 1999; Cast and Burke 2002). Figure 1 visualizes this process.

Conclusively, investment, self-esteem, and rewards are the outcomes of an identity-verification process, which arranges identities hierarchically based on successful or unsuccessful enactment. Higher-ranked identities become more important to the self-concept and, thus, have a larger influence on behavior. Issues arise due to the process of verification and the decisions about which identity to keep or dismiss and rank higher or lower. Corporate nomads, in particular, need to build an identity that balances individual lifestyle preferences and corporate work structures (Frick and Marx 2021). According to Prester et al. (2019), the work identity alone continuously balances the opposing poles of gaining professional autonomy and maintaining stability. Thus, it is important for individuals to receive social validation as a result of their actions and narratives that align with their identity formation process and provide assurance (Ashforth and Schinoff 2016, p.111).

In summary, issues of corporate nomads are a likely result of them trying to live up to the flexible and personal independence and, at the same time, achieving a sense of stability and establishing routines and structures within the organizational environment they are still embedded in.

## Research Design

### Data Collection

In this study, the research questions addressing the conceptualization of the corporate nomad archetype (RQ1) and possible identity issues of corporate nomads (RQ2) will be answered based on the results of fourteen qualitative interviews. The data collection period covered the time span from May 2021 to November 2021. The interviews represent cross-sectional data and provide a condensed way of collecting relevant information about the phenomenon (Bogner et al. 2009; Meuser and Nagel 2009).

The research team conducted and recorded the interviews in the international space via telecommunication means, such as phone calls, Skype, and Zoom. Two interviews were held in person in Bali with corporate nomads, as the island is one of the most important centers for expatriate workers worldwide. The experts, ranging from 23 to 53 years old, were contacted via expat forums online and in coworking spaces in Bali. Consequently, the purposely selected sample considered the experts’ natural and familiar environment, so that credible answers were expected. To generate an adequate amount of data, the sample comprises fourteen Western corporate employees that have at the time of the interview been abroad for at least twelve months (level-1 experts) or have gained at least 24 months of experience in remote work (level-2 experts) (see Table 2).

**Table 2:** Overview of the interview sample

Expert	Age	Job	Whereabout at the time of the interview	Interview Length
E1-L1	32	Online Marketing	Sweden	1:27:17
E2-L1	34	Consulting	Sweden	1:27:17
E3-L2	40	Marketing	Germany	40:23
E4-L1	33	Web-Design	Australia	49:30
E5-L2	34	Online Marketing	Germany	55:57
E6-L2	23	Social Media Manager	Germany	40:41
E7-L1	25	Finance	Ireland	33:09
E8-L1	32	Brand Manager	India	29:06
E9-L1	33	Finance	Colombia	26:32
E10-L1	26	Consulting	Bali	28:03
E11-L1	24	Finance	Bali	26:45
E12-L2	24	Online Marketing	Germany	29:23
E13-L1	53	IT-Development	Spain (Tenerife)	50:01
E14-L1	32	Marketing	Spain (Barcelona)	45:03

The level-2 experts were included in the sample to broaden the field of vision and avoid one-sidedness towards the phenomenon and to contrast the responses of level-1 corporate nomads. For the data collection, semi-structured, qualitative interviews as suggested by Meuser and Nagel (2009) were conducted by the research team in person or digitally with the help of a multi-page interview guide. The guide was designed based on the three main categories of identity theory: investment, self-esteem, and rewards (McCall and Simmons 1978), as well as the five dimensions identified in the literature: control, time, social environment, technology, and space (see Table 1). To obtain a rich understanding of the corporate nomad phenomenon, the interview guide mainly consists of open questions that focus on the description of corporate nomadism and allow for further questions during the interview. Open questions such as *“why did you choose this life/job?”* or *“do you give up certain things to make this life possible for yourself?”* provided respondents with a wide range of response options for detailed answers, evaluations, and their own opinions. The advantage of this type of interview is to guide the interviewees thematically without restricting them in their answers.

First, each interviewee was welcomed, the aim of the research was introduced, and “small talk” about life as a corporate nomad was held to build trust. In the next step, the expert described personal perceptions about being a corporate nomad or his or her experience with the phenomenon. If the answers were not detailed and profound enough, the interview guide offered a selection of possible follow-up questions to generate further information. Finally, the interview ended with an open space for last remarks and a short debriefing about the study.

### Data Analysis and Coding Process

To analyze the data, audio recordings were transcribed verbatim directly after the interviews, supported by the software Sonix.^2^ Subsequently, the transcriptions were translated into English if the interview has been held in German. A structuring qualitative content analysis was chosen as the method of analysis (Kuckartz 2014) as it is comprehensive, i.e., all material can be included if necessary. The content-structuring approach involves identifying and conceptualizing selected aspects of content in the material and describing and assigning the material with respect to the various facets of identity. Instead of applying deductive reasoning only, we followed the approach by Kuckartz (2014), who suggests following basic hermeneutic principles, i.e., instead of having a predefined system of categories, emerging concepts might change continuously. The first and second author performed deductive coding since the interview guide was set up with the help of dimensions from identity theory. To answer both research questions, the second step of the coding then involved a direct inductive assignment of the material to the different main categories to either classify the corporate nomad or assess possible issues corporate nomads are struggling with.

The content was summarized on a linguistic level through paraphrasing and labelling. In compliance with the content-structuring qualitative content analysis, we used a case/category table in which the different statements were filtered and assigned to the content-related categories. Thus, we were able to collect data that included feelings, emotions, and thoughts. An example excerpt from this table is shown in Table 3.

**Table 3:** Example case and category table

Main category	Subcategory	Statement	Summary	Expert
Investment	Job/Lifestyle Discrepancies	“…constant accessibility…”	Flexibility is good but company demands too much accessibility	E11-L1
Reward	Flexibility	“…a lot of freedom…”	Freedom through flexibility	E12-L2

## Results

### The Identity of Corporate Nomads

To obtain information about how the **level of control** is perceived by and divided between a corporate nomad and her employer, we looked at how task division is organized. Moreover, we considered habits of self-management and personal responsibility, and agreements with colleagues as well as clients. Most respondents report a liberal way of working.

When it comes to leadership, corporate nomads can work independently and do not have to follow strict rules and describe the hierarchies in their firms as rather flat. Furthermore, it was emphasized that personal responsibility and the willingness for personal growth is very important in this context (E3-L2).

*„So, the hierarchies are very flat; we are actually less managed rather in the sense that you are somehow a little bit steered in different directions maybe […] so that sometimes something is marked out or said yes maybe go in this or the other direction […], but actually the management interferes in almost nothing.“* (E5-L2)

In terms of the **allocation of time**, the respondents are mostly tied to core working hours or must agree upon appointments with colleagues and especially clients, where they have to be available. However, co-determination is part of the process as well. Mostly, flextime is reported with the possibility to consult within the team to arrange working hours in the event of possible interferences and according to personal preferences (E12-L2, E1-L1).

*“We have the core working time […] from nine to four […] in our team now I think we make it a bit flexible because there are also days where you must start later because you have […] two doctors appointments early and then, of course, I didn’t make it at nine. I think it’s good that it’s not seen quite so narrowly inside the team, and that’s important to me.”* (E4-L1)

Additionally, it is less about the number of working hours and more about the quality of the output (E7-L1, E3-L2, E1-L1).

*“Like I’m judged not based on the hours that I work for, based on the output of the projects that I’m working on. And so that’s like the biggest thing is that I should be available online, but I don’t have to be productive from nine to six.”* (E7-L1)

In the **social** dimension, the interviews revolved around how relationships develop and how work and non-work are related. Almost all interviewees speak of a positive mix or complete balance of working time and free time regarding the work-life balance. They also report that they are willing to go the extra mile for the company during stressful times because they know they will get time off afterward (E10-L1, E1-L1, E7-L1).

*“I wouldn’t want to change it. Of course, in some weeks, when there’s a bit more going on, you have a bit more stress. But you also have more relaxed weeks. And the advantage is that we have a very relaxed relationship at work, so if I have any important events in my private life or anything like that, I can always talk to my boss and get time off accordingly.”* (E11-L1)

The role of **technology** for organizing and managing work is characterized by a particularly large amount of work that is done with standardized software within the corporation. Thus, the devices for use are either fixed or must be agreed upon with the corporation (E3-L2, E7-L1).

*“I think that would be a bit difficult to implement in a company like this if everyone had different devices because we already use certain programs in a standardized way, and then somehow new software is introduced for everyone to use the telephone, for example, or something like that.”* (E5-L2)

As permanent employees in corporations, many interviewees have a stable social safety net, with benefits such as paid sick days, retirement plans, and insurance (E5-L2, E3-L2, E9-L1). If they do freelance work in addition, it is only for volunteer purposes, for the love of work or for personal development (E4-L1, E2-L1).

*“Because I am a permanent employee and not a freelancer, I am very well covered, just like employees are covered, paid sick days um, I also have a company pension plan.”* (E5-L2)

When it comes to the dimension of **space**, corporate nomads enjoy the freedom to work in a place of their own choice. There are typically no restrictions on the side of the employing corporation. The interviewees stated that they are free to choose whether they work at home (E3-L2, E12-L2) and free to decide whether and for how long they work abroad (E1-L1, E4-L1).

*“I currently work 100% remotely and choose my working environment according to the weather and my mood. So, when the weather is nice, i.e., the last few days, I’ve been working 100% outside in the garden. Yes, and otherwise I work in any room where I like it, where I have my peace and it is quiet, but I’m not tied down. For example, I could sit down in a café or whatever. Exactly, I can work anywhere I have internet access.”* (E3-L2)

*“We have a colleague who had the dream to travel through Europe with his campervan and work from anywhere. He also wanted to work only 3 days a week. That's what he proposed to us. And he is also a very good employee, [...] an A-player. Then you just have to fulfill all his wishes. We then decided at that time that he himself is responsible for making a successful project. He has now been in the van for a year.”* (E14-L2)

### Corporate Nomad Identity Issues

#### Issues of Investing in a Corporate Nomad Identity

An investment in a corporate nomad identity takes place when an individual actively performs actions or forms narratives that are consistent with this work ideal. Moreover, we consider it an investment when individuals waive or neglect certain circumstances, behaviors, or amenities to enable the corporate nomad lifestyle. Several aspects emerged from the data, of which the most important ones are discussed in the following. Three identity issues were salient in the investment category that relate to discrepancies between the nomadic lifestyle, job requirements, social life as well as a general lack of comfort and the constant risk of uncertainties.

Most mentioned problems were part of the ***(1) social separation*** corporate nomad identity issue. Being constantly abroad and not physically available can cause difficulties with social contacts at home including separation from family and friends as well as isolation from colleagues. Especially the contact with grandparents suffers from the distance due to differences in technical literacy (E7-L1).

*“Exactly, no longer participating in the family is the worst thing. But that’s what gnaws at you a bit. My grandma and grandpa celebrated their 60th wedding anniversary recently we weren’t there either. That’s the kind of thing that gnaws at you a bit.”* (E2-L1)

Common practices to compensate for the reduced social life includes working from co-working spaces where nomads can chat with like-minded people and establish new relationships (E3-L1, E4-L2). Another mentionable aspect is the impact of being a corporate nomad in a romantic relationship. For non-nomads it appears to be a deterring investment that must be accepted before deciding for a corporate nomad lifestyle. Even if a couple travels together as nomads there might be a constant social dependency on each other that impacts the relationship.

*“Because if one of you somehow can no longer cope abroad or loses his job or whatever can happen, then you are faced with the decision of how to proceed. So, I think there can be difficulties.”* (E12-L2)

In addition to that, corporate nomads report that they experience increased levels of ***(2) environmental distraction*** from the places they choose to work from, which increases the need for self-organization and discipline. Especially when coming to a new place, corporate nomads experience problems focusing on the job and not letting the lifestyle take overhand.

*“I actually think that I would have less motivation to work in the first few weeks, especially when you’re in a new place, you still want to experience a lot and do something, and you don’t want to be restricted by work. I think, therefore, the motivation first a little less. But I think, when you have found your way in your everyday life, it will come back again.”* (E12-L2)

Another corporate nomad identity issue emerges from ***(3) accessibility expectations***. Even though flexibility is a demanded prerequisite for many corporate nomads, they experience co-workers and managers expecting them to be constantly available. Due to often significant time changes, this is seen as a critical investment to be made.

*“I would say that the biggest disadvantage from my point of view is the, yes, constant accessibility, but at the same time, less contact with colleagues. Since you are working alone and if you are also alone at home, it could be that you feel lonely, and the little bit of social life is secondary.”* (E10-L1)

#### Issues of Developing Self-esteem as a Corporate Nomad

Self-esteem within the corporate nomad identity formation process is considered as the extent to which corporate nomads see themselves as self-efficient and able to manage obstacles they face. The following three identity issues related to this dimension of the identity verification process emerged throughout the interviews.

One central issue a corporate nomad might face arises from ***(4) holding multiple contradictory identities*** that could be difficult to unite. Concerning self-perception, a corporate nomad may have high expectations regarding occupying several roles, such as a caring parent and a time-flexible colleague.

*“I used to think that I would have to give up work completely to be a mother.”* (E7-L1)

*“[…] that you don't see the family. You don't just talk to your family on the phone for three or four hours. Especially when there are six people sitting on the other side, brothers and sisters with their partners and parents and grandparents. You can't have a quiet conversation there either.”* (E1-L1)

However, the interviewees mentioned that the experiences they gained through a nomadic lifestyle led to ***(5) disproportionate personal growth*** in comparison to their old corporate environment. Interviewees reported that they were able to develop their personalities or the ability to familiarize themselves better with new tasks. Thus, upcoming issues could be transferred into developing new skills such as solution-oriented working and problem-solving. Further examples are one interviewee overcoming his shyness and another one feeling strong enough to combine parenting with a nomadic lifestyle (E4-L1, E7-L1).

*“So, I’m more confident that I’ll be able to do both if I want to or if I want to work part-time or if I want to work flexibly and be able to drop my kids it’s not going to impact my career, so I think that’s been the biggest thing that I know that I won’t have to sacrifice what I’m doing to be a parent.”* (E7-L1)

We found that corporate nomads develop strong independency and autonomy while working remotely. If problems and questions occur, they ask colleagues or challenge themselves to grow from them.

*“Yes, I often get new tasks. Then I take time with a colleague who has already done the tasks. And we have a meeting via teams and share our screens and she then tries to familiarize me with the task. And then I still have time to work through it myself. And yes, that’s how it works for us. I actually see more of an advantage there, because I can take my time and make notes on it myself, rather than having someone sitting next to me in the office looking over my shoulder.”* (E10-L1)

To what extent this finding is an identity issue depends largely on leadership and how this disproportionate growth is nurtured or suppressed.

However, in the pursuit to increase self-efficacy, a ***(6) flexibility paradox*** emerges in some cases. For instance, one corporate nomad was complaining about too flexible working hours and not being able to say “no” if the supervisor asked for overtime work (E11-L1). In contrast, another corporate nomad was hoping for more flexibly arrangeable working times so that work could be done even at weekends (E10-L1). The corporate nomads we interviewed were divided in their opinion about independent and dependent work schedules and at that point were often not able to successfully carry out desired actions themselves to enhance self-efficacy.

*„I would perhaps like to have a bit more of a timeline, because it’s actually very, very flexible for us. That means my boss calls me today, says you can work later, and then I work later. And I say, so I often find it difficult to say no. So, most of the time, I jump, and maybe it would be advantageous to get a little bit more of a guideline. I´d prefer being stricter with myself and say no sometimes”* (E11-L1)

#### Issues of Experiencing Rewards as a Result of a Corporate Nomad Identity

With rewards, we mean experiences that encourage further identity verification attempts. Those attempts, in turn, are made once individuals gain intrinsic and extrinsic gratification. The following part presents issues in the process of corporate nomads trying to reinforce their identities.

As the first issue in this category, we identified a ***(7) savings-salary-gap*** in the realm of external rewards. Overall, the interviewees expressed satisfaction salary-wise and mentioned that there were no changes once they went abroad while still working for the same company. However, there were different opinions about future corporate nomad salaries being higher than those of the local population.

*“This is a peculiar topic. We haven't actually had this problem yet, except with me. I sort of have a geo advantage now. I have my Amsterdam salary and I live in Barcelona. There the conditions are of course quite different (laughs). And if they had said they would cut my salary, I don't know if I would have stayed there. I am still the same person.”* (E14-L1)

One interviewee is even expecting higher salaries as a corporate nomad since companies will save office-related expenses per employer (E11-L1).

*“I could imagine that other employers might actually pay more, because you also gain experience abroad and, once again, the company has greater added value through its internationality.”* (E12-L2)

During our interviews, we identified that satisfaction in terms of salary can be achieved by simply being able to pursue the lifestyle of a corporate nomad. Pursuing personal goals and changing lifestyle and mindset provided many corporate nomads with overall work satisfaction so that the salary level did not play a key role anymore.

*“If you compare it to other strict and bigger agencies, I would probably earn more but that’s just necessarily not where my priorities are.”* (E5-L2)

With reference to their supervisors some interviewees mentioned that through commitment to work and being trusted, they felt strongly connected to work and the company and could get work done autonomously (E7-L1). They further appreciated additional virtually organized get-togethers, which created a familiar and friendly atmosphere.

*“As I have just said, there is a weekly or every two weeks a meeting, where everyone then tells how it is going. I had for example my birthday, there was a meeting, a birthday meeting or yes, something like that is already endeavored there.”* (E10-L1)

The corporate nomads were able to identify differences in motivation between remote and regular work. While some were dedicated to living as corporate nomads for as long as possible, some others wanted to quit once they decided to settle down. Overall, the corporate nomads tend towards the attitude that they have crossed a ***(8) point of no remote-return*** and they prefer remote work so much that they would consider quitting their job if their employer took this freedom away from them.

*“So, I would probably never want to do a 100 percent office again in my life. If it were a model that said okay, two days, three days a week in the office and the rest of the time at home or you work or depending on how far away the office is, of course. But primarily, I would say no, if then only partially.”* (E3-L2)

The advantages of having no strict schedules, individual organization, and being able to move in between locations were mentioned frequently.

*“I would choose remote because the flexibility is really pleasant. Especially when you’re young, you’re not bound to go to the office every day and always have this daily routine. When you’re at home and work at home, you can organize everything a bit more individually, whatever you want to do. Yes, I just like it better that I’m not tied to a certain place.”* (E12-L1)

A major disadvantage some interviewees pointed out is that some group work preferences are eliminated by corporate nomadism. For some, less face-to-face interactions inhibit efficient collaboration within group work assignments. Therefore, corporate nomads that prefer face-to-face interaction experience ***(9) collaboration constraints*** when working in groups. One interviewee thought that he could get work done faster if colleagues were around (E8-L1). Another corporate nomad, in turn, mentioned that productivity was higher at home because there was more distraction in the office.

*“In the office it can be very easy to get distracted like with people and chatting and all this kind of stuff so I definitely find like I’m probably more productive when I’m at home however I probably take fewer breaks so maybe it’s less stainable in the long term but I’m no I would say it’s very different. Would say in the office I’m definitely motivated but I definitely am more distracted by people.”* (E7-L1)

## Discussion

### Towards a Conceptualization of the Corporate Nomad Archetype

This paper constitutes a qualitative approach exploring the identity of corporate nomads and how this emerging knowledge worker archetype can be defined. As the first step of this discussion, we provide an extended definition of the corporate nomad archetype. This definition is based on the knowledge we retrieved from previous literature (see Table 1) and the results of our empirical inquiry. The extended definition of the corporate nomad archetype reads as follows.

**Table:** 

**Corporate nomads are permanent employees working location-independently by leveraging information technology.** They differ from digital nomads in that they benefit from a robust social safety net and adhere to core working hours and standardized technology. They differ from 9–5 corporate workers in that they create their lifestyle and work environment according to individual preferences and democratic decisions in concert with superiors and co-workers	

**Fig. 2:**
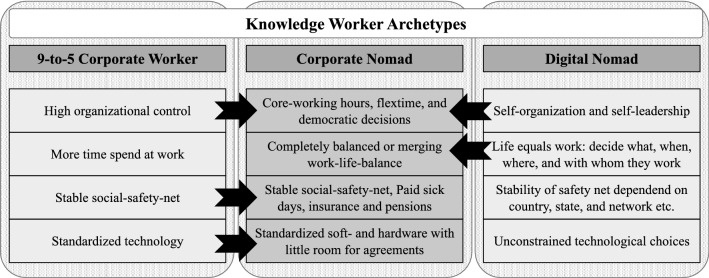
Comparison between knowledge worker archetypes, adapted from Wang et al. (2020)

En route to conceptualizing the corporate nomad, we have discovered that a synthesis between the two existing knowledge worker archetypes – the 9-to-5 corporate worker and the digital nomad – is an accurate but rough description of this emerging archetype. Our work unearthed the aspects and dimensions in which the corporate nomad assumes characteristics of each of the known archetypes. Here, we build on the work of Wang et al. (2020). However, in comparison to their ideals of knowledge worker archetypes (‘knowmad’ vs. ‘cyborg’), our aim was to equip the IS literature with an additional concept that represents the contemporary but overlooked corporate nomad phenomenon and enriches the bandwidth of knowledge worker archetypes. Figure 2 illustrates which aspects of the corporate nomad archetype refer to the previously known characteristics of the 9-to-5 corporate worker and the digital nomad (indicated by arrows).

The identity of the corporate nomad, for example, borrows from the ideal of the 9-to-5 corporate worker in terms of the social safety net. This is interesting because not long ago, Prester et al. (2019) found that one of the biggest drawbacks for digital nomads was a missing social safety net. In terms of technology use, the corporate nomad defers to the technology standard provided by their firm. However, slight deviations can take place and individual agreements about the choice of technology are possible for some (E3-L2, E7-L1, E5-L2). Be it software or hardware – corporate nomads need to ensure that company-wide and customer communication and cooperation can be carried out seamlessly (E3-L2, E7-L1). However, this is by no means a negative connotation because corporate nomads feel supported and empowered by that technology. As far as organizing work is concerned, a synthesis of digital nomad and corporate 9-to-5 work can be observed. Corporate nomads are free to work from wherever they want – living a location-independent lifestyle on the shoulders of heavy technology use – but are bound to core working hours and meetings with clients, even though they are often given flextime and arrangements with their own teams and superiors. This is attributed to the permanent employment of corporate nomads and the associated structures of those companies (E1-L1, E12-L2). Ultimately, the social dimension of a corporate nomad identity has a stronger tendency towards the digital nomad ideal. Corporate nomads report utmost satisfaction with their work-life balance and, in some cases, do not distinguish between life and work due to their interlinked passions for work and travel (E10-L1, E1-L1, E7-L1). In comparison to existing knowledge worker archetypes, e.g., as suggested by Wang et al. 2020, the corporate nomad archetype is unparalleled because it combines characteristics of two conceptions of work that so far have not been in alignment (Kong et al. 2019). The adoption of the corporate nomad concept in research and practice, however, may yield some unintended consequences. The societal implications of the mainstreamization of digital nomadism only gradually surface now in the form of competitive digital nomad visa schemes, fiscal problems, or environmental imbalances (Jiwasiddi et al. 2022). Institutionalizing corporate nomadism, in turn, may pose major challenges in terms of policy, governance, legal affairs, or fairness. While many corporate nomad practices still fly below the radar of regulation, canny IS research that addresses design or policy implications of corporate nomadism is needed now to help organizations maneuvering through this transformation of socio-spatial configurations of knowledge work.

### Resolving Emergent Corporate Nomad Identity Issues

To answer RQ2, we need to recall the identity verification process as laid out by identity theory (Burke and Stets 2009; McCall and Simmons 1978). Issues of corporate nomads result from ***trying to create flexible and personal independence while adhering to stability, routines, and structures*** within the organizational environment. Managing the combination of corporate and nomadic values within a corporate nomad’s self-concept can lead to discrepancies due to differing preferences, poor implementation or adherence to their respective institutional logistics, and a mismatch between their worldviews (Frick and Marx 2021). The empirical analysis of this work helped to identify these discrepancies. To solve the identified corporate nomad identity issues, it is crucial to consider the related dimensions of the identification process, i.e., the dimensions investments, self-esteem, and rewards of the identity verification process (Stets and Serpe 2013). Understanding those underlying bonds allows us to conceptually derive explicit recommendations for resolving the corporate nomad identity issues in relation to the knowledge worker dimensions. Table 4 summarizes the corresponding dimensions of the identification process as well as the knowledge worker dimensions in relation to the identified corporate nomad identity issues.

**Table 4:** Overview of corporate nomad identity issues related to identity verification and knowledge work dimensions

Identity verification outcome	Knowledge work dimension	Corporate nomad identity issues
Investment	Social/space	Social separation Spatial distance to work contacts Spatial distance to family members and other social contacts
	Space	Environmental distraction Self-responsibility to create a productive work environment outside of corporate premises
	Time	Accessibility expectations Shifted working hours (e.g., across time zones) and missing detachment from onsite work boundaries
Self-esteem	Social	Holding multiple contradictory identities e.g., corporate 9–5 worker vs. digital nomad; corporate high achiever vs. stay-home parent
	Social/control	Disproportionate personal growth Developing skills and autonomy that surpass the requirement of previous work arrangement
	Control/time	Flexibility paradox Increased freedom while feeling the urge to “do more”
Rewards	Control	Savings-salary gap Potential savings of the employer are not invested in corporate nomad employee
	Control/space	Point of no remote-return Non-acceptance of future onsite work arrangements by corporate nomads
	Technology/space	Collaboration constraints Loss of informal exchange of information Gain of distraction-free work sessions

### Investments in a Corporate Nomad Identity

Investments occur mainly when corporate nomads shape their environment due to structures and rules that they are embedded in (McCall and Simmons 1978). It appeared that some of the actual privileges were perceived as too excessive or as not enough. Regarding the issue *social separation,* Lee et al. (2019) suggest that the organization of social and work life boundaries is one central factor in creating a *private place* for work. To verify the identity of a valuable employee, the corporate nomad needs to create a *private place* that eliminates the issue of *environmental distraction.* Moreover, different time zones may evoke *accessibility expectations* to fulfill team members’ needs across the globe (Wilk 2016). Those issues may further enhance social separation from the partner or family by being less available for their social needs. Furthermore, being in a relationship with a person with a non-nomadic identity may burden a corporate nomad with the fear of failing a long-distance relationship (E12-L2). It appears to be a challenge for both organizations and employees to find work arrangements that suit both parties without limitation. Corporate nomads that were most satisfied stated that they have semi-flexible core working hours without time tracking and a strong basis of trust. To resolve the issues related to investment dimensions, organizations need to establish a culture that employees relieve from the pressure of being constantly accessible and support individual preferences (Frick and Marx 2021; Lee et al. 2019). This relief action might be supported by transparently communicating the needs and requirements of the organization by coordinating core-working hours and flextime.

### Self-esteem from Identifying as a Corporate Nomad

However, the verification of a professional identity might contradict the social identity of being a close friend or family member. When the presence of both identities overlaps, due to poor work-life balance or overwhelming professional requirements, *holding multiple contradictory identities* may violate the self-esteem of a corporate nomad because the nomad cannot sufficiently meet the expectations of both identities (Cast and Burke 2002). However, the impact of organizational processes on identity might be crucial considering the control over collaboration partners and provided technology (Whitley et al. 2014). The empirical analysis of our interview data suggests that the corporate nomad archetype is subject to great diversity. Motivators of pursuing corporate nomadism vary from economic to familial to ideological. Nevertheless, most of our interviewees claim to find (self-)assurance in investing in a corporate nomad identity because it planes the contradictions between digital nomadism and corporate work (Kong et al. 2019). By performing extraordinary identity work (Brown 2015), most corporate nomads aim for or achieve *identity equanimity*, i.e., resolving tensions not only between corporate and digital nomad identities but also between other conflicting identities. For example, identity work towards corporate nomadism may result in combining the identities of being a corporate high achiever and being a parent in constant physical proximity to one’s children. It may also relieve the tension between living a nomadic lifestyle and weaving a net of stable income, social security, or retirement planning (Prester et al. 2019). The identity work of corporate nomads is often emotional (e.g., *“who had the dream to travel” (E14-L2), “I would probably never want to do a 100 percent office again in my life “ (E3-L2), “I would have to give up work completely to be a mother” (E7-L1)),* which is in line with previous literature on identity work (Winkler 2018). The aspired equanimity as a result of identity work, in this sense, calms the emotional turbulence that is caused by identity conflicts.

The distance to a physical office could also affect working performance through the *flexibility paradox* in one way or the other. Being location independent may affect the perceived self-esteem, which is enhanced when a corporate nomad sees herself as self-efficient and capable of managing faced obstacles (Cast and Burke 2002). In comparison with 9–5 corporate workers, corporate nomads might perceive *disproportionate personal growth* by solving upcoming problems and developing new skills that the typical 9-to-5 corporate worker would not face the same way. Thus, organizations should consider and support the different ways of solving tasks and gaining new skills through their organizational control. For example, our findings indicate that connecting corporate nomads with 9-to-5 corporate workers could create synergy.

In that context, some corporate nomads prefer being independent and are convinced that the autonomy of individually chosen working hours enhances productivity and leads to the main positive driver of stress-free work habits. However, the findings reveal that some corporate nomads have the feeling of not being able to say “no” regarding overtime work. They feel the urge to prove to supervisors and colleagues that they are working and not only traveling. The interviewees seem to think increased work performance means acceptance by colleagues and supervisors (E12-L2). This shows that, on the one hand, some organizations establish the feeling of high organizational control, while others overstrain their corporate nomads by demanding a high degree of self-organization. Thus, it is crucial to carefully balance the individual control and time dependencies of corporate nomads to successfully integrate this type of knowledge worker in prevailing organizational structures.

### Intrinsic and Extrinsic Rewards from Identifying as a Corporate Nomad

By stepping into independency and leaving behind a settled lifestyle, the interviewed corporate nomads had to make additional investments to obtain their identity. However, employees may only accept this additional effort if they can expect certain desired rewards. The efforts made by corporate nomads are all made to live a nomadic lifestyle while being corporate employees. The individuals we interviewed were neither content with a traditional corporate identity nor an identity that is typically associated with a highly independent and self-employed digital nomad. We found that for most of our interviewees, investing in combining nomadic values with corporate values led to enhanced self-esteem and increased rewards (compared to the rewards they received from their prior work ideals). Both extrinsic and intrinsic rewards could be found to contribute to the identity formation process of corporate nomads. A distinct extrinsic reward is a salary that the organization itself can control and that may encourage further identity-verification attempts (Cast and Burke 2002; Stryker and Burke 2000). Regarding the *savings-salary-gap*, some corporate nomads expressed that they receive the same salary independent of their working style while other had to make compromises salary-wise. The satisfaction with their lifestyle might suppress a low remote worker salary in the short term but possible financial difficulties in the future mean a premature termination of a nomadic lifestyle for some of the interviewees. Considering the dimension of control, organizations need to transparently communicate potential salary-gaps that evolve from a corporate nomad lifestyle, as E11-L1 expects even higher salaries as companies might save office-related costs by the employment of corporate nomads. The author team is also aware of the idea that companies might consider paying less to corporate nomads in comparison to their non-nomad co-workers. Our results suggest that if companies would do so, they risk losing (or not winning) employees with the intention of living as corporate nomads. This will result in a competitive disadvantage. Providing intrinsic or extrinsic rewards is a way to support the formation of a positive identity of corporate nomads, thus establishing a strong connection to the organization. However, organizations need to consider that embracing corporate nomad models, in the short term, prioritizes individual preferences, which can lead to organization growth in the long term. This is problematic if organizational values contradict the aspired identity of a corporate nomad. A potential threat to identity (Mirbabaie et al. 2022) could be the removal or change of the corporate nomad model in the organization. This threat to the corporate nomad identity may evoke identity protection behavior resulting in a job change. At this stage, the way of life of a corporate nomad chooses becomes a high-value intrinsic reward. The corporate nomad reached the *point of no remote-return* that probably an increased salary could not outdo. This issue may emphasize the importance of creating a private place for corporate nomad work that is not physically bound to a distinct location but rather a working environment and life model that suits the individual employee’s identity.

This study and its implications come with limitations. It should be noted that the collected sample represents corporate nomads and does not include individuals that represent the 9–5 corporate worker or a digital nomad archetype. However, we heavily rely on literature that has done precisely that and, therefore, are the first study to present a purposeful sample of corporate nomads. Moreover, the perception of the corporate nomad phenomenon might be biased due to pandemic circumstances and office restrictions. It might have been more salient in the period of our data collection. The validity of the findings could be strengthened with a larger overall sample.

## Conclusion

This paper examined nomadic behaviors of organizational employees by means of qualitative interviews. The analysis of the interview data resulted in a definition and conceptualization of the corporate nomad knowledge worker archetype and the identification of nine issues that impact the identity formation of corporate nomads: social separation, environmental distraction, accessibility expectations, holding multiple contradictory identities, disproportionate personal growth, a flexibility paradox, savings-salary gaps, the point of no remote-return, and collaboration constraints. By identifying these concepts, we have added knowledge to the field of IS and the future of work and challenge the prevailing conception that a home office in a private place is bound to one physical location. As a response to the potential identity issues of corporate nomads, we suggest that the principle of *identity equanimity* promises remedy from those issues. This is because identity work of corporate nomads is largely a product of attempting to resolve contradictions of pre-defined identities. Moreover, the discussion of our results provides guidance for organizations and corporate nomads on how to institutionalize corporate nomadism for both individual and organizational gain.

This study provides a unique perspective on the corporate nomad phenomenon and aims to provide food for thought when it comes to rethinking prevalent conceptions of knowledge work and its components such as home office. Further research should consider the management perspective of experts who are experienced in leading corporate nomads. Moreover, a dedicated study could examine the role of IT and certain character traits for the identity verification process of corporate nomads. Case studies of organizations deploying corporate nomad work in different industries would further strengthen this research stream in the IS literature. Our findings suggest that corporate nomadism is just one manifestation of challenging the status quo. We encourage fellow IS researchers to consider researching alternative work-life-technology concepts such as corporate nomadism to be able to reflect upon predominant organizational conduct and create auspicious visions of the future of work – including one’s own.

## Footnotes

1 We adopt the term ‘ideal’ from the work of Wang et al. (2020), who use this *“Weberian concept of the “ideal type” (Idealtypus). An ideal type draws attention to particular social phenomena by articulating them as an abstract analytic archetype, accentuating certain characteristics, elements, and points of view (Weber 1904).”* (p. 1381).

2 https://sonix.ai/.
